# MicroRNAs overexpressed in ovarian ALDH1-positive cells are associated with chemoresistance

**DOI:** 10.1186/1757-2215-6-18

**Published:** 2013-03-22

**Authors:** Young Tae Park, Ju-yeon Jeong, Mi-jung Lee, Kwang-il Kim, Tae-Heon Kim, Young-do Kwon, Chan Lee, Ok Jun Kim, Hee-Jung An

**Affiliations:** 1 Department of Emergency Medicine, Kosin University, Busan, South Korea; 2 Department of Pathology, CHA University, Sungnam, 463-712, South Korea; 3 Institute for Clinical Research, CHA University, Sungnam, South Korea; 4 Department of Gynecologic Oncology, College of Medicine, CHA University, Sungnam, South Korea

**Keywords:** Ovarian cancer, Cancer stem cell, ALDH1, microRNA, Chemoresistance

## Abstract

**Background:**

Ovarian carcinoma is the leading cause of cancer death worldwide among gynecological malignancies, and the majority of cases are related with recurrence and chemoresistance. Cancer stem cells (CSCs) are believed to be one of the causes of recurrent or chemoresistant ovarian cancer, and microRNAs are regulatory molecules newly implicated to control a variety of cellular processes, including CSCs. Therefore, we identified ovarian CSC-specific microRNAs and investigated their clinicopathological implication in ovarian carcinomas.

**Methods:**

We isolated ALDH1 (+) cell population using the Aldefluor assay, and examined the differential expression pattern of miRNAs between ALDH1 (+) and ALDH1 (−) cells using a high-throughput microRNA microarray. We further investigated the expression patterns of differentially expressed miRNAs in human ovarian cancer samples using the real-time reverse transcription-polymerase chain reaction and analyzed their clinical impact in patients with ovarian cancer.

**Results:**

We found that high ALDH1 expression was associated with chemoresistance in *in vitro* and *ex vivo* samples (p = 0.024). We identified six miRNAs, including miR-23b, miR-27a, miR-27b, miR-346, miR-424, and miR-503, overexpressed in ALDH1 (+) cells, and they were significantly upregulated in chemoresistant ovarian cancer cells (1.4 ~ 3.5-fold) and tumor samples (2.8 ~ 5.5-fold) compared with chemosensitive group. Upregulation of ALDH1 (p = 0.019) and miR-503 (p = 0.033) correlated with high clinical stage, and upregulation of miR-27a was related with distant metastasis (p = 0.046) in patients with ovarian cancer.

**Conclusion:**

Our findings indicate that ALDH1 is a useful marker for enriching ovarian CSCs, and high expression of ALDH1 and its related miRNAs, particularly miR-23b, miR-27b, miR-424, and miR-503, are significantly implicated in chemoresistance and tumor progression in ovarian cancer.

## Background

Ovarian carcinoma is the leading cause of gynecologic cancer deaths [1], and > 70% of patients with advanced stage develop a recurrence within 5 years [2]. Despite multimodality treatment, many patients with advanced disease become refractory to conventional chemotherapeutic agents [3], resulting in recurrence and death.

Emerging evidence suggests that cancer stem cells (CSCs) represent the most tumorigenic and chemotherapy-resistant cells within a heterogeneous tumor mass [4,5]. CSCs are characterized by their ability to self-renew, modulate, and balance differentiation according to the genetic background and environmental stimuli [6]. CSCs can survive conventional treatments and become recurrent tumors that are more chemoresistant and more aggressive [7]. However, the CSC regulatory mechanisms at the molecular level are poorly understood. The recent discovery of microRNAs (miRNA) have opened a field of gene regulation implicated in tumorigenesis and CSC modulation [8].

MiRNAs, noncoding regulatory RNAs of 21–25 nucleotides [9], are critical regulators of post-transcriptional gene expression. MiRNAs are transcribed as long RNA precursors (primary miRNAs) that are processed to yield mature miRNAs of approximately 25 nucleotides in length by Drosha-Pasha/DGCR8 and Dicer. Mature miRNAs are incorporated into the RNA-induced silencing complex and then target the 3′ untranslated region (3′-UTR) of a specific mRNA by base pairing, leading to translational repression or mRNA degradation [10]. MiRNAs have been predicted to regulate the expression of up to 70% of human genes, implying a potential role for miRNAs in the regulation of nearly every genetic pathway [11,12]. Taken together, understanding the regulatory role of miRNAs in CSCs may lead to a better understanding of the molecular events involved in chemoresistance and could lead to the development of a new therapeutic target.

In this study, we isolated the ALDH1 (+) cell population enriched CSCs using the Aldefluor assay, and examined the differential expression pattern of miRNAs between ALDH1 (+) and ALDH1 (−) cells using a high-throughput microRNA microarray to identify miRNAs regulating ovarian CSCs. We further investigated the expression patterns and their clinical impact of differentially expressed miRNAs in human ovarian cancer samples using the real-time reverse transcription polymerase chain reaction (qRT-PCR).

## Methods

### Cell lines and tumor samples

The human ovarian carcinoma cell line SKOV3 was obtained from the American Type Culture Collection (Manassas, VA, USA). Paclitaxel (PTX)-resistant cell lines (SKpac) were produced from the parent cell line (SKOV3) by continuous exposure of a stepwise, escalating concentration of PTX from an IC_50_ of 10% to 1000% over a period of 12 months. Seven different sublines (SKpac-8, -10, -11, -12, -13, -16, and −17) were generated. SKpac cells were 365.5-fold more resistant to PTX (IC_50_ = 10.21 μM, 7.59 μM, 6.77 μM, 6.57 μM, 8.69 μM, 8.38 μM, and 5.32 μM, respectively) than that of the SKOV3 cell line (IC_50_ = 22 nM). All cell lines were maintained in 5A medium (Gibco/Invitrogen, Carlsbad, CA, USA) with 10% fetal bovine serum (FBS) (Invitrogen), 100 U/mL penicillin, and 50 μg/mL streptomycin in a humidified atmosphere containing 5% CO_2_ at 37°C. The primary tumor cells were obtained at the time of surgery from patients who had undergone oophorectomy for ovarian serous carcinoma. Tumors were mechanically dissected into single-cell suspensions and incubated at 37°C for 1 hour in Ca^2+^/Mg^2+^-free PBS containing 50 U/ml collagenase A (Roche, Pleasanton, CA, USA). The enzymatic reaction was blocked by adding RPMI medium (Gibco/Invitrogen) containing 10% FBS. Cells were reacted with BerEP4-coated magnetic Dynal beads (Invitrogen) for 30 min to select epithelial cells and then cultured with RPMI medium containing 10% FBS, 1% penicillin-streptomycin, and 10 μg/ml of endothelial cell growth factor (Invitrogen).

Thirty-four ovarian carcinomas were obtained at the time of surgery from patients who had undergone oophorectomies for ovarian epithelial tumors at the CHA Bundang Medical Center. The samples were immediately frozen in liquid nitrogen and stored at −80°C. Tumor cells comprised nearly 80% of frozen section tissue.

Clinical and pathological data were retrieved from clinical databases including the original pathology reports with histological grade and tumor stage. A histopathological examination of these samples was performed in the Department of Pathology according to the criteria of the International Federation of Gynecology and Obstetrics grading system and the World Health Organization classification. The patient’s samples were devided into two groups according to the responsiveness to the first-line chemotherapy. Based on the NCCN guidelines, intrinsically chemoresistant tumors were defined as those with persistent or recurrent disease within 6 months after the initiation of first-line taxol-platinum-based combination chemotherapy. Chemosensitive tumors were classified as those with a complete response to chemotherapy and a platinum-free interval of >6 months. This study was approved by the Ethical Committee of the CHA Bundang Medical Center, and informed consent was obtained from each patient prior to surgery. The clinicopathological data are summarized in Table 1.

**Table 1:** Clinicopathological characteristics of patients with ovarian serous carcinoma (n = 34)

		**Chemosensitive**	**Chemoresistant**
Age (yrs)		51.5 ± 18.5	64.5 ± 18.5
Stage	I/II	4(11.76%)	0(0.00%)
	III/IV	15(44.12%)	15(44.12%)
LN	Negative	9(26.47%)	5(14.71%)
	Positive	10(29.41%)	10(29.41%)
Metastasis	Negative	14(41.17%)	10(29.41%)
	Positive	5(14.71%)	5(14.71%)
Total		19(55.88%)	15(44.12%)

### ALDEFLUOR assay and fluorescence-activated cell sorting (FACS)

The ovarian cancer cell line (SKOV3), chemoresistant sublines (SKpac-12, 16, and 17), and primary ovarian tumor cell suspensions were counted and ALDH enzymatic activity was detected using the ALDEFLUOR assay kit (StemCell Technologies Vancouver, BC, Canada) as described by the manufacturer. Half of the cell/substrate mixture in each sample was treated with 50 mM diethylaminobenzaldehyde (DEAB), and the cells were incubated for 40 minutes. Gating was established using propidium iodide (PI) exclusion for viability, and ALDEFLUOR/DEAB treated cells were used to define negative gates. FACS was performed with 1 × 10^6^ cells using the BD FACS Aria III (Becton Dickinson, Franklin Lakes, NJ, USA) under low pressure in the absence of UV light. FACS data were analyzed using BD FACS Diva software V6.1.3.

### RT-PCR analysis of stem cell marker genes

Semiquantitative RT-PCR was performed to assess the amount of stemness-related gene mRNA in ALDH1(+) cells compared with that in ALDH1(−) cells. Total RNA was extracted from ALDH1(+) or ALDH1(−) cells acquired by FACs sorting using Trizol reagent (Invitrogen). RNA (1 μg) was reverse-transcribed in a total volume of 20 μl using the SuperScript First-strand Synthesis system (Invitrogen). The primers for these genes are listed in Table 2. The PCR products were loaded on a 2% agarose gel, the ethidium bromide stained gel image was digitalized using the Molecular Imager Gel Doc™ XR System (Bio-Rad, Hercules, CA, USA), and calculated by densitometry. GAPDH was used as the reference gene to normalize mRNA amounts between samples. Results are presented as relative mRNA expression of each gene to that of GAPDH mRNA.

**Table 2:** Primer sequence of stemness-related genes

**Gene**	**Primer sequence**
Notch3	F: 5′- TCT TGC TGC TGG TCA TTC TC-3′
	R: 5′-TGC CTC ATC CTC TTC AGT TG-3′
Oct4	F: 5′-GGA AAG GCT TCC CCC TCA GGG AAA GG-3′
	R: 5′-AAG AAC ATG TGT AAG CTG CGG CCC-3′
NAC1	F: 5′-CCA GAC ACT GCA GAT GGA GA-3′
	R: 5′-AAG CTG AGG ATC TGC TGG AA-3′
c-Kit	F: 5′-GGC ATC ACG GTG ACT TCA AT-3′
	R: 5′-GGT TTG GGG AAT GCT TCA TA-3′
BMI1	F: 5′-TTA CCT GGA GAC CAG CAA GT-3′
	R: 5′-CAT TAG AGC CAT TGG CAG CA′3′
Nanog	F: 5′-AGA AGG CCT CAG CAC CTA C-3′
	R: 5′-GGC CTG ATT GTT CCA GGA TT-3′
Sox-2	F: 5′-GCG CGG GCG TGA ACC AG-3′
	R: 5′-CGG CGC CGG GGA GAT ACA-3′
GAPDH	F: 5′-ACC ACA GTC CAT GCC ATC AC-3′
	R: 5′-TCC ACC ACC CTG TTG CTG TA-3′

### Spheroid formation assay

The FACS-sorted ALDH1 (+) or ALDH1(−) cells were plated in wells of an ultra-low attachment surface 6-well culture plate (Corning, Acton, MA, USA) at a density of 3,000 cells/ml in serum-free DMEM/F12 medium (Invitrogen) supplemented with 20 ng/ml epidermal growth factor (Invitrogen), 10 ng/ml basic fibroblast growth factor (Sigma-Aldrich, St Louis, MO, USA), 0.4% bovine serum albumin (Sigma-Aldrich), and 5 μg/mL insulin (Sigma-Aldrich). Spheroid formation (each with 50–100 cells/sphere) was assessed 12 days after seeding.

### miRNA microarray

miRNA expression profiles of the FACS-isolated ALDH1(+) or ALDH1(−) cells from four PTX-resistant SKpac cell lines (SKpac-16-#1,-16 #2, -17#1, and −17#2) were determined with an Affymetrix GeneChip® miRNA array to select candidate miRNAs associated with ALDH1. The samples were prepared according to the manufacturer’s instructions. Total miRNA was isolated using TriZol reagent (Invitrogen), and RNA quality was assessed with an Agilent 2100 Bioanalyzer using the RNA 6000 Nano Chip (Agilent Technologies, Amstelveen, The Netherlands). One μg of total RNA was used as input for the labeling reaction, and labeled miRNA was hybridized to the array for 16 hours at 48°C and 60 rpm as described in the protocol. After hybridization, the chips were stained and washed in a Genechip Fluidics Station 450 (Affymetrix) and scanned with a Genechip Array scanner 3000 7G (Affymetrix).

### Validation of ALDH1 and selected miRNA expression levels by qRT-PCR

We performed real-time qRT-PCR for ALDH1 and six selected miRNAs that showed significantly altered expression in ALDH1(+) cells compared with ALDH1(−) cells to validate the ALDEFLUOR assay and the microarray data. The qRT-PCR for ALDH 1 was conducted with ALDH1(+) or ALDH1(−) cells from SKpac-12, 16, and 17 cells. One μg of total RNA was converted to cDNA with the SuperscriptIII First-strand Synthesis System (Invitrogen) and qRT-PCR was conducted with specific primers and a probe for ALDH1. For miRNAs, one μg of total RNA was reverse transcribed to cDNA using stem-loop specific RT-primers of mature miRNAs and a TaqMan MicroRNA Reverse Transcription Kit (Applied Biosystems, Foster City, CA, USA). The qRT-PCR was performed with a Taqman Universal Master Mix (Applied Biosystems) using the Bio-Rad CFX96 Real-Time PCR Detection System (Bio-Rad). All PCR reactions were run in triplicate, and gene expression relative to GAPDH (for ALDH1) or RNU48 (for miRNAs) was calculated using the comparative threshold method (2^-ΔΔCt^). Results for the ALDH1 (+) cells are expressed as fold-change relative to ALDH1 (−) cells.

We also conducted the qRT-PCR in various ovarian cancer cell lines (SKOV3, SKpac-8, 10, 11, 12, 13, 16, and 17) and 34 ovarian serous carcinoma samples including 15 chemoresistant and 19 chemosensitive carcinomas to assess the association ALDH1 and ALDH1(+)-related miRNA expression with chemoresistance. The results for the cancer cell lines are expressed as fold-changes of chemoresistant SKpac cells relative to the level of parent SKOV3 cells. The results for carcinoma samples are expressed as fold-change relative to the mean level of three normal ovarian surface epithelial cells.

## Results

### Subpopulations of ALDH1 (+) cells in the ovarian cancer cell lines

We examined the population of ALDH1(+) cells, a potential cancer stem cell marker, in various ovarian cancer cell lines (SKOV3, A2780, and OVCAR 3), chemoresistant sublines (SKpac-12,-16,-17, A2780pac, and A2780cis), and primary tumor cells (SCN-1–7). The ALDH1(+) cells varied considerably among ovarian cancer cell lines with a range of 0.9–17.2% and with a range of 0.4–4.0% among the primary cancer cells (Table 3).

**Table 3:** Population of ALDH1 (+) cells as determined by the ALDEFLUOR assay in various human ovarian cancer cell lines and primary cells

**Cells**	**% ALDH1 (+) cell**
**SKOV3**	0.9 ± 0.2
**Skpac**	4.46 ± 0.71
**A2780**	1.66 ~ 7
**A2780pac**	7.96
**A2780cis**	2.25
**Ovcar3**	17.16
**SCN 1**	0.4
**SCN 2**	0.5
**SCN 3**	0.9
**SCN 4**	3.96
**SCN 5**	1.9
**SCN 6**	1.35
**SCN 7**	4.05

SCN, primary ovarian cancer cells.

**Figure 1:**
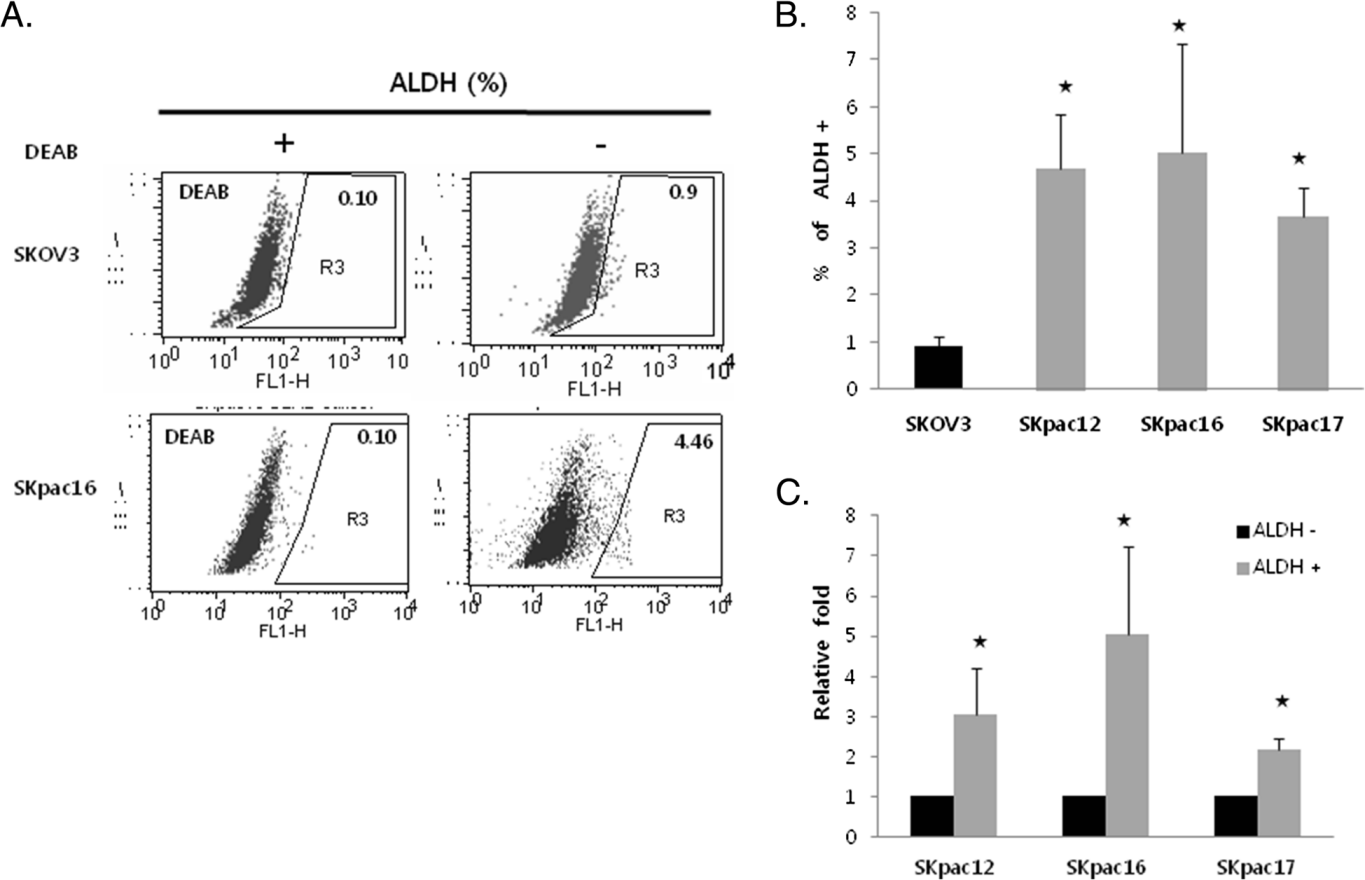
**Population of ALDH1(+) cells in ovarian cancer cells. A**. ALDH1 enzymatic activity in chemosensitive SKOV3 and chemoresistant SKpac16 cells as determined by the Aldefluor assay. DEAB is a specific inhibitor of ALDH1 and was used to confirm gating areas. **B**. A comparison of the percentage of ALDH1(+) in SKOV3 cells and chemoresistant SKpac-12, -16, and −17 cells. **C**. ALDH1 mRNA expression in FACS-sorted ALDH1 (+) and (−) cells. All data are representative of at least two independent experiments. (*p < 0.05).

The ALDH 1(+) population percentage by FACS analysis in chemoresistant SKpac cells (4.46 ± 0.71%) increased significantly (Figure 1A,B) compared with that in parental SKOV3 cells (0.9 ± 0.2%), suggesting an association between ALDH 1(+) cells and chemoresistance.

We performed qRT-PCR for ALDH1 mRNA to confirm whether FACS-sorted ALDH1 (+) cells showed increased ALDH1 expression. The FACS-sorted ALDH1(+) population showed 2–5 fold higher expression of ALDH1 mRNA compared with that in the ALDH1(−) population (Figure 1C).

### RT- PCR analysis of stemness markers in ALDH1(+) and ALDH1 (−) cells

**Figure 2:**
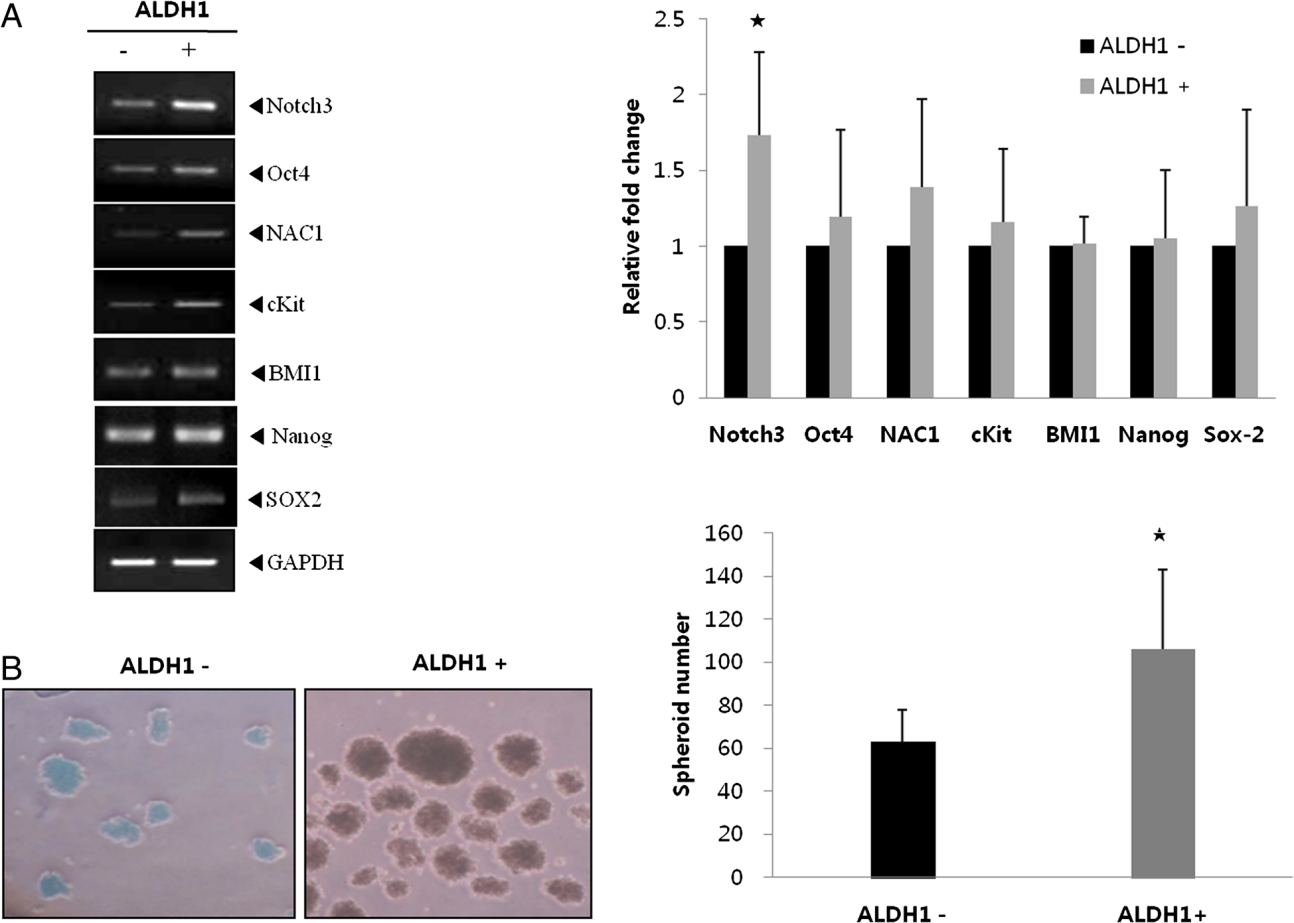
**ALDH1(+) cells show stem cell properties. A**. mRNA expression of stemness-associated genes, particularly Notch 3, increased in ALDH1(+) cells compared with that in ALDH1(−) cells. The graph represents the relative fold expression of band density normalized to that of GAPDH (*p < 0.05). **B**. The spheroid formation assay revealed an increased number of spheroids in ALDH1(+) cells. The graph presents the average spheroid formation in ALDH1(+) and ALDH1(−) cells (*p < 0.05). All data are representative of at least two independent experiments.

The stem cell phenotype of the ALDH1(+) cells was confirmed by expression of several stemness-associated markers for Notch3, Oct-4, NAC1, c-Kit, BMI-1, Nanog, and SOX2 using semiquantitative RT-PCR. The mRNA expression of Notch3 (1.73-fold) was significantly higher (*t*-test, p = 0.04) in ALDH1(+) cells than that in ALDH1(−) cells (Figure 2A). Oct-4 (1.19-fold), NAC1 (1.39 -fold), and Sox2 (1.26-fold) mRNA levels tended to be elevated in ALDH1(+) cells compared with those in ALDH1(−) cells, but the difference was not significant. BMI-1 (1.02-fold), c-Kit (1.16-fold), and Nanog (1.05-fold) mRNAs were not differentially expressed.

### Spheroid formation assay of ALDH1 (+) and ALDH1 (−) cells

We compared the spheroid formation capability between ALDH1 (+) and ALDH 1(−) cells to test the stem cell-like property. ALDH1(+) cells showed increased size and number (106 ± 37) of spheroids compared with ALDH1(−) cells (63 ±15) (Figure 2B) (*t*-test, p = 0.05).

### Implication of ALDH1-positivity for chemoresistance and clinicopathological parameters

**Figure 3:**
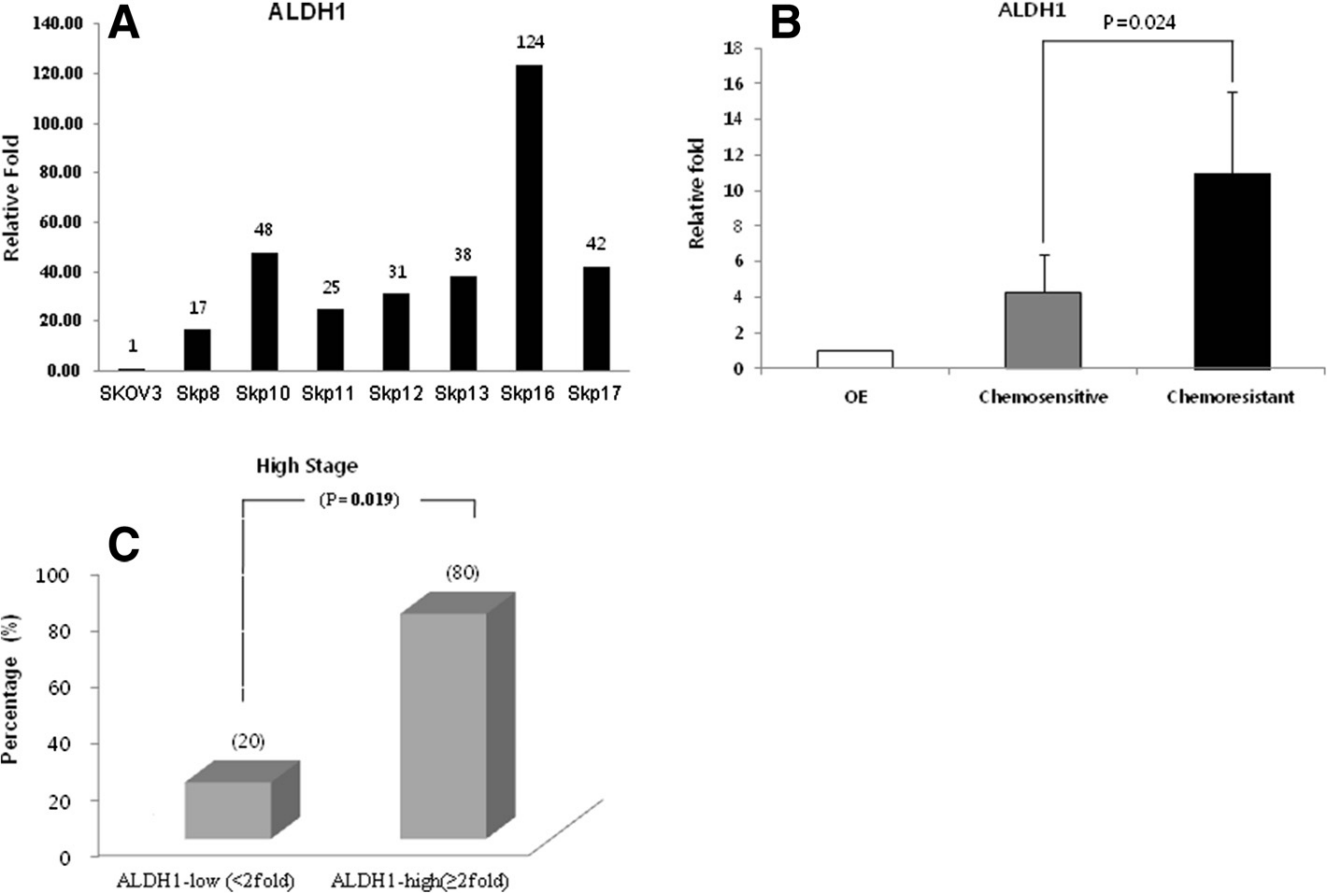
**ALDH1-positivity was associated with chemoresistance in ovarian cancer cells and tumor tissues. A**. ALDH1 mRNA expression by qRT-PCR in various chemoresistant SKpac sublines. Relative expression levels are normalized to parent SKOV3 cells. **B**. ALDH1 mRNA expression by qRT-PCR in chemoresistant ovarian cancer tissues was significantly higher than that in the chemosensitive group. **C**. To verify the role of ALDH1 in ovarian cancer, we analyzed the correlation between ALDH1 mRNA expression and clinicopathological parameters, including clinical stage, lymph node metastasis, and distant metastasis. Patients were classified into high (≥2-fold) and low (<2-fold) expression groups for comparison. High ALDH1 mRNA expression was significantly (p = 0.019) correlated with advanced clinical stage.

We examined ALDH1 mRNA expression in ovarian cancer cells and 34 cases of ovarian cancer tissue samples using qRT-PCR. The expression levels of various chemoresistant SKpac cells were compared with those of the chemosensitive SKOV3 parent cell line. ALDH1 mRNA expression in chemoresistant SKpac cells was markedly elevated (17 ~ 124-fold) compared with that in SKOV3 (Figure 3A). The ALDH1 mRNA expression level relative to that of normal epithelial cells was calculated and compared between chemosensitive and chemoresistant groups in the human cancer tissue samples. The relative expression level of ALDH1 mRNA (Figure 3B) in the chemoresistant group of human cancers (11-fold) was significantly higher (*t*-test, p = 0.024) than that of the chemosensitive group (4.29-fold).

To assess the role of ALDH1 in ovarian cancer, we analyzed the relationship of ALDH1 mRNA expression with clinicopathological parameters, including clinical stages, lymph node metastasis and distant metastasis. For comparison, patients were classified into high (≥2-fold) and low (<2-fold) ALDH1 expression groups. The high expression of ALDH1 mRNA was significantly (p = 0.019) associated with advanced clinical stage (Figure 3C). The ALDH 1 expression was not correlated with lymph node or distant metastases.

### microRNA expression patterns in ALDH1(+) cells assessed by microarray

**Figure 4:**
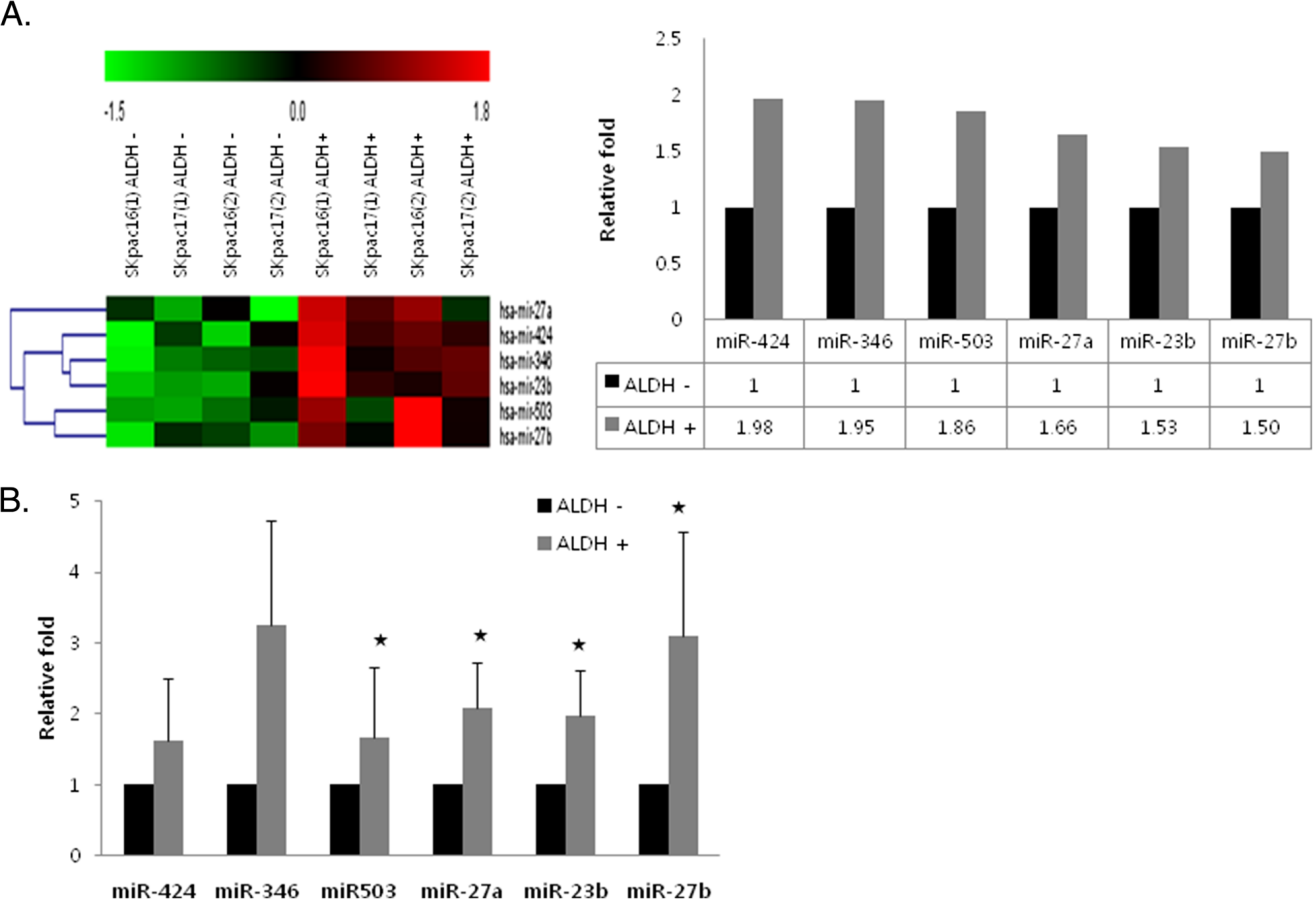
**MicroRNAs differentially overexpressed in ALDH1 (+) cells compared with ALDH1 (−) cells. A**. Hierarchical clustering of miRNA expression profiles by miRNA microarray. Unsupervised hierarchical clustering analysis of miRNAs that exhibited a statistically significant (p < 0.05) increase or decrease in ALDH1 (+) cells compared to ALDH1 (−) cells. The miRNA expression level is color-coded. **B**. Validation of candidate miRNAs expression by qRT-PCR. The bar graph shows the expression of each microRNA in flow cytometry-sorted ALDH1 (+) cells relative to the average expression level in ALDH1 (−) cells. Representative experiments repeated three times (*p < 0.05).

We performed the Affymetrix GeneChip microarray analysis containing 4,560 human precursor and mature miRNA oligonucleotide probes to characterize the miRNA expression profile of ALDH1 (+) cells. As a result, six miRNAs were differentially overexpressed more than 1.5-fold in ALDH1 (+) cells compared with that in ALDH1 (−) cells (Table 4, Figure 4A) (miR-424 [1.98-fold], miR-346 [1.95-fold], miR-503 [1.86-fold], miR-27a [1.66-fold], miR-23b [1.53-fold], and miR-27b [1.50-fold]). No miRNAs were downregulated in ALDH1 (+) cells.

**Table 4:** MicroRNAs significantly altered (>1.5 fold) in ALDH1(+) cells compared with ALDH1 (−) cells in ovarian cancer

**Name**	**Absolute fold change**	**P-value**	**Chromosome**	**Putative target genes**
hsa-miR-424	1.98	0.02	Xq26.3	WEE1, CDCA4, USP15, LATS2, HIPK2
hsa-miR-346	1.95	0.01	10q23.2	PTPN18, LIF
hsa-miR-503	1.86	0.05	Xq26.3	CDCA4
hsa-miR-27a	1.66	0.03	19p13.13	GSPT1, CDS1, TAB3, SFRP1, MDM4, PRKCB, SESN2, FOSB, SMAD2
hsa-miR-23b	1.53	0.02	9q22.32	APAF1, PPP2R5E, PPP1CB, PPIF, REPS2, CHUK
hsa-miR-27b	1.50	0.03	9q22.32	GSPT1, CDS1, TAB3, PRKCB

The target genes presented are predicted using the TargetScan (http://www.targetscan.org) and PicTarVert (http://pictar.mdc-berlin.de) softwares.

We compared expression of six differentially expressed miRNAs between ALDH1(+) and ALDH1(−) cells using real-time RT-PCR to validate the microarray results. As a result, miR-424 (1.62-fold), miR-346 (3.25-fold), miR-503 (1.66-fold), miR-27a (2.08-fold), miR-23b (1.98-fold), and miR-27b (3.09-fold) were upregulated in ALDH1 (+) cells relative to ALDH1 (−) cells (Figure 4B).

### Implication of ALDH1(+)-associated miRNAs in chemoresistance and clinicopathological parameters of ovarian cancer

**Figure 5:**
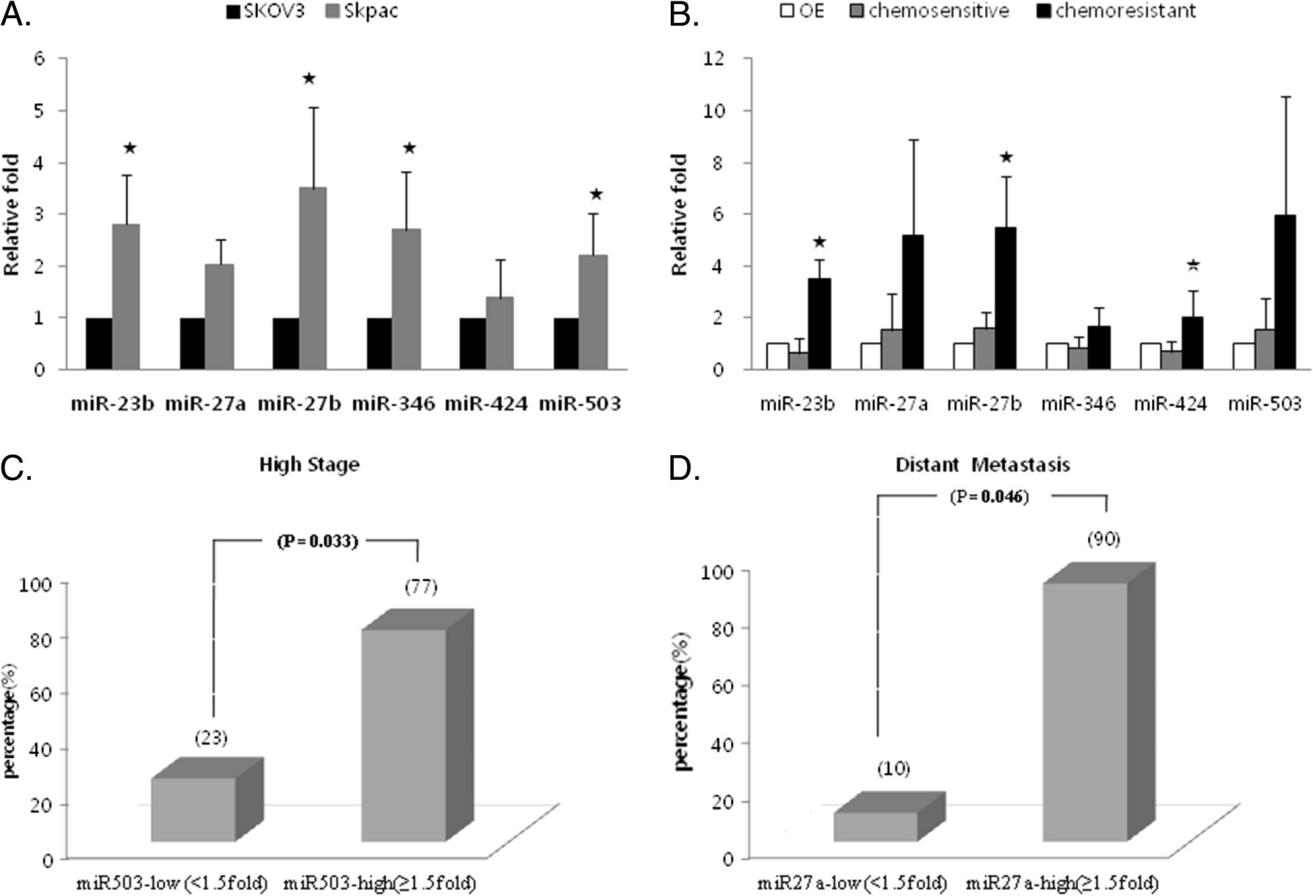
**ALDH1 (+)- related miRNAs are associated with chemoresistance in ovarian cancer cells and tumor tissues. A**. A comparison of miRNA expression in SKOV3 and chemoresistant SKpac cells. miRNAs-23b, -27b, -346, and −503 were significantly upregulated in SKpac cells compared with SKOV3 parent cells. **B**. Expression of ALDH1 (+)-related miRNAs by qRT-PCR in chemosensitive and chemoresistant ovarian cancer tissues. Relative expression levels were normalized to those of normal epithelial cells. Expression levels of miRNAs-23b, -27b, and −424 were significantly upregulated in the chemoresistant group compared with those in the chemosensitive group. Representative experiments repeated three times (*p < 0.05). **C**. High expression of miR-503 significantly correlated (p = 0.033) with advanced clinical stage (stage III and IV). **D**. Upregulation of miR-27a was associated with distant metastasis (p = 0.046).

We examined the expression of these miRNAs in ovarian cancer cells and 34 cases of ovarian cancer tissue samples using qRT-PCR to assess the impact of six ALDH1(+)-related miRNAs on chemoresistance in ovarian cancers. The expression levels of various chemoresistant SKpac cells were compared with those of the chemosensitive SKOV3 parent cell line. Among the miRNAs examined, the expression levels of miR-23b (2.8-fold, p = 0.039), miR-27b (3.5-fold, p = 0.007), miR-346 (2.7-fold, p = 0.02), and miR-503 (2.2-fold, p = 0.049) were significantly higher than those in SKOV3 cells (Figure 5A).

The expression levels of miRNAs in the human cancer tissue samples relative to those of normal epithelial cells were calculated and compared between the chemosensitive and chemoresistant groups. The expression levels of miR-23b (3.5-fold vs. 0.6-fold p = 0.037), miR-27b (5.5-fold vs. 1.6-fold, p = 0.040) and miR-424 (2.0-fold vs. 0.7-fold, p = 0.047) were significantly higher in the chemoresistant group than those in the chemosensitive group (Figure 5B).

We analyzed the correlation between the expression of these miRNAs and clinicopathological parameters, including clinical stage, lymph node metastasis, and distant metastasis to verify the role of ALDH1(+)-associated miRNAs in ovarian cancer. Patients were classified into high (≥1.5-fold) and low (<1.5-fold) expression groups. High expression of miR-503 (Figure 5C) was significantly associated (p = 0.033) with advanced clinical stage (stage III and IV), and upregulation of miR-27a (Figure 5D) was related to distant metastasis (p = 0.046). No other miRNAs were associated with the clinicopathological parameters.

## Discussion

CSCs comprise a very small proportion (0.01–1.0%) of malignant tumors but are believed to be the source of recurrent tumors and of resistance to chemotherapy and radiotherapy due to their ability to survive conventional treatments, which usually target rapidly dividing cells [7,13]. Therefore, identifying CSCs and their molecular phenotype might serve to develop a new therapeutic strategy for lethal human cancers including ovarian carcinoma. Indeed, several studies focusing on identifying and characterizing ovarian CSCs have been reported recently [14-16]. These studies suggested several markers including CD44, CD117, CD133, and ALDH1 as possible ovarian CSC markers. Among them, ALDH1 is considered to be a consistent ovarian CSC marker identified in all ovarian cancer cell lines and primary human cancer samples [15]. Therefore, we used ALDH1 as a putative ovarian CSC marker in the present study and confirmed that ALDH1(+) cells were enriched with ovarian CSCs by demonstrating an increased expression of stem cell markers and increased spheroid formation ability compared with those in ALDH1(−) cells.

A growing body of evidence indicates that miRNAs regulate most human genes in crucial biological processes including tumorigenesis, progression, and therapy resistance. MiRNAs also seem to be involved in controlling CSC self-renewal and differentiation, according to more recent studies reporting differentially expressed miRNAs in CSCs of various human cancers [17-19]. Recently, deregulation of some miRNAs, such as miR-200a, miR-199a, and miR-214, were reported in CD133(+) or CD44(+) ovarian CSCs [20]. However, only limited data are available regarding microRNA expression profiles in ovarian CSCs. Therefore, we identified ovarian CSC-specific miRNAs using high-throughput microRNA microarray and assessed the association of expression of differentially expressed miRNAs with chemoresistance in ovarian cancer.

We found that six miRNAs, including miR-23b, miR-27a, miR-27b, miR-346, miR-424, and miR-503, were significantly overexpressed in CSC-enriched ALDH1(+) cells using miRNA microarray and qRT-PCR. We demonstrated that these miRNAs and ALDH1 increased significantly in chemoresistant SKpac sublines compared with those in the chemosensitive SKOV3 cell line, suggesting that these miRNAs and ALDH1 are associated with ovarian cancer chemoresistance. To validate the relationship of ALDH1 and ALDH1-related miRNAs with chemoresistance, we further studied the expression of ALDH1 and these miRNAs in human ovarian cancer samples. Consistent with the results of the ovarian cell lines, ALDH1 mRNA expression in the chemoresistant group was significantly higher (2.5-fold) than that in the chemosensitive group, and the expression of miR-23b, miR-27b, and miR-424 was significantly upregulated in the chemoresistant group. Upregulation of ALDH1 and miR-503 correlated with high clinical stage, and upregulation of miR-27a was correlated with distant metastasis.

Our result showing the correlation between high ALDH1 expression and chemoresistance agrees with some other recent breast and ovarian cancer studies [21-23]. The present study also demonstrated that PTX-resistant subclones (SKpac cells) were more enriched in ALDH1(+) cells compared with their parental SKOV3 cells. We also found higher ALDH1 expression in chemoresistant ovarian cancer tissues compared with that in the chemosensitive group. It is possible that clonal selection of intrinsic ALDH1(+) cells by chemotherapy may increase ALDH1 expression in chemoresistant cancer cells and may allow the acquisition of chemoresistance in residual cancers. Chang et al. [24] reported a correlation between low ALDH1 expression and advanced cancer stage and response to cisplatin treatment. However, Chang et al. included heterogeneous subtypes of ovarian cancer, including the endometrioid and mucinous types, and used immunohistochemical staining with a tissue microarray, which may not represent the characteristics of the whole tumor. More recent studies [15,23] have suggested an association between high ALDH1 expression and advanced cancer stage and poor response to chemotherapy, consistent with our results. Taken together, targeting ALDH1 in ovarian cancer may be a new strategy to treat chemoresistant and advanced ovarian carcinoma.

In the present study, miR-23b, miR-27b, and miR-424 were significantly upregulated in the chemoresistant human cancer group among six miRNAs differentially overexpressed in ALDH1(+) cells. MiR-23b has been reported to function as a renal cancer oncogene [25], similar to ovarian cancer seen in the present study, whereas it functions as a tumor suppressor in hepatocellular carcinoma [26]. The introduction of pre-miR-27b stimulates invasion in breast cancer cells by targeting the ST14 tumor suppressor [27], and is upregulated in glioma cells and tumor tissues [28]. However, it is downregulated in lung cancer tissue [29]. A recent study for miRNAs expression signatures in tumorigenesis [30] indicated that miR-424 was associated with angiogenesis, and that miR-23b and miR-27b were upregulated in metastatic stage. They also demonstrated that miR-424 were up-regulated in metastases compared with primary tumors in both mice and human pancreatic neuroendocrine tumors. Another study for non-small cell lung cancers demonstrated that up-regulation of miR-424 was related with poor prognosis [31]. On the other hand, it was reported that low expression of miR-424 was positively correlated with advanced clinical stage, lymph node metastasis and other poor prognostic parameters in cervical cancers [32]. Taken together, the oncogenic function of miR-23b, miR-27b, and miR-424 seems to be cell type- and context-specific.

The upregulation of miR-27a didnot show a statistically significant correlation with chemoresistance in the validation with paclitaxel-resistant SKpac sublines and patient’s samples in this study. However, a previous study reported that miR-27a was upregulated in paclitaxel-resistant ovarian cancer cell line A2780/Taxol as compared with its parental line A2780 [33]. In the present study, high expression of miR-27a was related with distant metastasis suggesting its role on the progression of ovarian cancer.

The function of miR-503 in tumor development and progression remains unresolved except for a report demonstrating its regulation of metastatic function in hepatocellular cancer cells [34]. Therefore, we searched predicted target genes associated with apoptosis and tumor suppression via web-based computational programs such as Target Scan (http://www.targetscan.org) and PicTar-Vert (http://pictar.mdc-berlin.de) to predict the role of miR-503 implicated in chemoresistance and tumor progression. CDCA4, a putative miR-503 target gene with a high target score, participates in the regulation of cell proliferation mainly through the E2F/pRB pathway [35]. Further studies are necessary to determine whether miR-503 induces cancer cell growth or migration/invasion, and whether CDCA4 is a direct target gene of miR-503, as well as to define the oncogenic function of miR-503 in this subset of ovarian cancers.

## Conclusions

We demonstrated in this study that ALDH1 is a marker for enriching ovarian CSCs, and that high ALDH1 expression is associated with chemoresistance and high clinical stage. Six miRNAs, including miR-23b, miR-27a, miR-27b, miR-346, miR-424, and miR-503 were overexpressed in ALDH1(+) cells, and significantly implicated in chemoresistance in ovarian cancers. Upregulation of miR-503 correlated with high clinical stage, and upregulation of miR-27a was related with distant metastasis in patients with ovarian cancer.

## Competing interests

The authors declare that they have no competing interests.

## Authors’ contributions

YTP drafted the manuscript. JYJ carried out the FACS analysis and cell culture. MJL carried out the molecular studies. KIK participated in the sequence alignment and drafted the manuscript. THK performed the statistical analysis. CL performed the interpretation of data. OJK participated in its design and coordination. HJA conceived of the study, and participated in its design and coordination and helped to draft the manuscript. All authors read and approved the final manuscript.

## References

[B1] Jemal A Thomas A Murray T Thun M Cancer statistics, 2002 CA Cancer J Clin 2002 52 23 47 10.3322/canjclin.52.1.23 11814064

[B2] Ozols RF Bundy BN Greer BE Fowler JM Clarke-Pearson D Burger RA Mannel RS DeGeest K Hartenbach EM Baergen R Phase III trial of carboplatin and paclitaxel compared with cisplatin and paclitaxel in patients with optimally resected stage III ovarian cancer: a gynecologic oncology group study J Clin Oncol 2003 21 3194 3200 10.1200/JCO.2003.02.153 12860964

[B3] McGuire WP Hoskins WJ Brady MF Kucera PR Partridge EE Look KY Clarke-Pearson DL Davidson M Cyclophosphamide and cisplatin compared with paclitaxel and cisplatin in patients with stage III and stage IV ovarian cancer N Engl J Med 1996 334 1 6 10.1056/NEJM199601043340101 7494563

[B4] Dean M Fojo T Bates S Tumour stem cells and drug resistance Nat Rev Cancer 2005 5 275 284 10.1038/nrc1590 15803154

[B5] Rosen JM Jordan CT The increasing complexity of the cancer stem cell paradigm Science 2009 324 1670 1673 10.1126/science.1171837 19556499 PMC2873047

[B6] Clarke MF Fuller M Stem cells and cancer: two faces of eve Cell 2006 124 1111 1115 10.1016/j.cell.2006.03.011 16564000

[B7] Reya T Morrison SJ Clarke MF Weissman IL Stem cells, cancer, and cancer stem cells Nature 2001 414 105 111 10.1038/35102167 11689955

[B8] Calin GA Croce CM MicroRNA signatures in human cancers Nat Rev Cancer 2006 6 857 866 10.1038/nrc1997 17060945

[B9] Ambros V The functions of animal microRNAs Nature 2004 431 350 355 10.1038/nature02871 15372042

[B10] Bartel DP MicroRNAs: genomics, biogenesis, mechanism, and function Cell 2004 116 281 297 10.1016/S0092-8674(04)00045-5 14744438

[B11] Lewis BP Burge CB Bartel DP Conserved seed pairing, often flanked by adenosines, indicates that thousands of human genes are microRNA targets Cell 2005 120 15 20 10.1016/j.cell.2004.12.035 15652477

[B12] Esquela-Kerscher A Slack FJ Oncomirs - microRNAs with a role in cancer Nat Rev Cancer 2006 6 259 269 16557279 10.1038/nrc1840

[B13] Hambardzumyan D Becher OJ Holland EC Cancer stem cells and survival pathways Cell Cycle 2008 7 1371 1378 10.4161/cc.7.10.5954 18421251

[B14] Burgos-Ojeda D Rueda BR Buckanovich RJ Ovarian cancer stem cell markers: prognostic and therapeutic implications Cancer Lett 2012 322 1 7 10.1016/j.canlet.2012.02.002 22334034 PMC4431611

[B15] Silva IA Bai S McLean K Yang K Griffith K Thomas D Ginestier C Johnston C Kueck A Reynolds RK Aldehyde dehydrogenase in combination with CD133 defines angiogenic ovarian cancer stem cells that portend poor patient survival Cancer Res 2011 71 3991 4001 10.1158/0008-5472.CAN-10-3175 21498635 PMC3107359

[B16] Zhang S Balch C Chan MW Lai HC Matei D Schilder JM Yan PS Huang TH Nephew KP Identification and characterization of ovarian cancer-initiating cells from primary human tumors Cancer Res 2008 68 4311 4320 10.1158/0008-5472.CAN-08-0364 18519691 PMC2553722

[B17] Ji J Yamashita T Budhu A Forgues M Jia HL Li C Deng C Wauthier E Reid LM Ye QH Identification of microRNA-181 by genome-wide screening as a critical player in EpCAM-positive hepatic cancer stem cells Hepatology 2009 50 472 480 10.1002/hep.22989 19585654 PMC2721019

[B18] Shimono Y Zabala M Cho RW Lobo N Dalerba P Qian D Diehn M Liu H Panula SP Chiao E Downregulation of miRNA-200c links breast cancer stem cells with normal stem cells Cell 2009 138 592 603 10.1016/j.cell.2009.07.011 19665978 PMC2731699

[B19] Yu F Yao H Zhu P Zhang X Pan Q Gong C Huang Y Hu X Su F Lieberman J Song E Let-7 regulates self renewal and tumorigenicity of breast cancer cells Cell 2007 131 1109 1123 10.1016/j.cell.2007.10.054 18083101

[B20] Nam EJ Lee M Yim GW Kim JH Kim S Kim SW Kim YT MicroRNA profiling of a CD133+ spheroid-forming subpopulation of the OVCAR3 human ovarian cancer cell line BMC Med Genomics 2012 5 18 10.1186/1755-8794-5-18 22643117 PMC3480901

[B21] Tanei T Morimoto K Shimazu K Kim SJ Tanji Y Taguchi T Tamaki Y Noguchi S Association of breast cancer stem cells identified by aldehyde dehydrogenase 1 expression with resistance to sequential Paclitaxel and epirubicin-based chemotherapy for breast cancers Clin Cancer Res 2009 15 4234 4241 10.1158/1078-0432.CCR-08-1479 19509181

[B22] Croker AK Goodale D Chu J Postenka C Hedley BD Hess DA Allan AL High aldehyde dehydrogenase and expression of cancer stem cell markers selects for breast cancer cells with enhanced malignant and metastatic ability J Cell Mol Med 2009 13 2236 2252 10.1111/j.1582-4934.2008.00455.x 18681906 PMC6512388

[B23] Landen CN Jr Goodman B Katre AA Steg AD Nick AM Stone RL Miller LD Mejia PV Jennings NB Gershenson DM Targeting aldehyde dehydrogenase cancer stem cells in ovarian cancer Mol Cancer Ther 2010 9 3186 3199 10.1158/1535-7163.MCT-10-0563 20889728 PMC3005138

[B24] Chang B Liu G Xue F Rosen DG Xiao L Wang X Liu J ALDH1 expression correlates with favorable prognosis in ovarian cancers Mod Pathol 2009 22 817 823 19329942 10.1038/modpathol.2009.35 PMC2692456

[B25] Liu W Zabirnyk O Wang H Shiao YH Nickerson ML Khalil S Anderson LM Perantoni AO Phang JM miR-23b targets proline oxidase, a novel tumor suppressor protein in renal cancer Oncogene 2010 29 4914 4924 10.1038/onc.2010.237 20562915 PMC4398970

[B26] Salvi A Sabelli C Moncini S Venturin M Arici B Riva P Portolani N Giulini SM De Petro G Barlati S MicroRNA-23b mediates urokinase and c-met downmodulation and a decreased migration of human hepatocellular carcinoma cells FEBS J 2009 276 2966 2982 10.1111/j.1742-4658.2009.07014.x 19490101

[B27] Wang Y Rathinam R Walch A Alahari SK ST14 (suppression of tumorigenicity 14) gene is a target for miR-27b, and the inhibitory effect of ST14 on cell growth is independent of miR-27b regulation J Biol Chem 2009 284 23094 23106 10.1074/jbc.M109.012617 19546220 PMC2755715

[B28] Chen L Li H Han L Zhang K Wang G Wang Y Liu Y Zheng Y Jiang T Pu P Expression and function of miR-27b in human glioma Oncol Rep 2011 26 1617 1621 21922148 10.3892/or.2011.1458

[B29] Yanaihara N Caplen N Bowman E Seike M Kumamoto K Yi M Stephens RM Okamoto A Yokota J Tanaka T Unique microRNA molecular profiles in lung cancer diagnosis and prognosis Cancer Cell 2006 9 189 198 10.1016/j.ccr.2006.01.025 16530703

[B30] Olson P Lu J Zhang H Shai A Chun MG Wang Y Libutti SK Nakakura EK Golub TR Hanahan D MicroRNA dynamics in the stages of tumorigenesis correlate with hallmark capabilities of cancer Genes Dev 2009 23 2152 2165 10.1101/gad.1820109 19759263 PMC2751988

[B31] Donnem T Fenton CG Lonvik K Berg T Eklo K Andersen S Stenvold H Al-Shibli K Al-Saad S Bremnes RM Busund LT MicroRNA signatures in tumor tissue related to angiogenesis in non-small cell lung cancer PLoS One 2012 7 e29671 10.1371/journal.pone.0029671 22295063 PMC3266266

[B32] Xu J Li Y Wang F Wang X Cheng B Ye F Xie X Zhou C Lu W Suppressed miR-424 expression via upregulation of target gene Chk1 contributes to the progression of cervical cancer Oncogene 2013 32 976 987 10.1038/onc.2012.121 22469983

[B33] Li Z Hu S Wang J Cai J Xiao L Yu L Wang Z MiR-27a modulates MDR1/P-glycoprotein expression by targeting HIPK2 in human ovarian cancer cells Gynecol Oncol 2010 119 125 130 10.1016/j.ygyno.2010.06.004 20624637

[B34] Zhou J Wang W Analysis of microRNA expression profiling identifies microRNA-503 regulates metastatic function in hepatocellular cancer cell J Surg Oncol 2011 104 278 283 10.1002/jso.21941 21495032

[B35] Hayashi R Goto Y Ikeda R Yokoyama KK Yoshida K CDCA4 is an E2F transcription factor family-induced nuclear factor that regulates E2F-dependent transcriptional activation and cell proliferation J Biol Chem 2006 281 35633 35648 10.1074/jbc.M603800200 16984923

